# Peripheral nerve injury results in a biased loss of sensory neuron subpopulations

**DOI:** 10.1097/j.pain.0000000000003321

**Published:** 2024-08-15

**Authors:** Andrew H. Cooper, Allison M. Barry, Paschalina Chrysostomidou, Romane Lolignier, Jinyi Wang, Magdalena Redondo Canales, Heather F. Titterton, David L. Bennett, Greg A. Weir

**Affiliations:** a School of Psychology and Neuroscience, University of Glasgow, Glasgow, United Kingdom; b Nuffield Department of Clinical Neurosciences, University of Oxford, Oxford, United Kingdom

**Keywords:** Sensory neuron, Neuron death, Transgenic reporter line, Neuropathic pain, Nerve injury

## Abstract

There is a rich literature describing the loss of dorsal root ganglion (DRG) neurons following peripheral axotomy, but the vulnerability of discrete subpopulations has not yet been characterised. Furthermore, the extent or even presence of neuron loss following injury has recently been challenged. In this study, we have used a range of transgenic recombinase driver mouse lines to genetically label molecularly defined subpopulations of DRG neurons and track their survival following traumatic nerve injury. We find that spared nerve injury leads to a marked loss of cells containing DRG volume and a concomitant loss of small-diameter DRG neurons. Neuron loss occurs unequally across subpopulations and is particularly prevalent in nonpeptidergic nociceptors, marked by expression of Mrgprd. We show that this subpopulation is almost entirely lost following spared nerve injury and severely depleted (by roughly 50%) following sciatic nerve crush. Finally, we used an in vitro model of DRG neuron survival to demonstrate that nonpeptidergic nociceptor loss is likely dependent on the absence of neurotrophic support. Together, these results profile the extent to which DRG neuron subpopulations can survive axotomy, with implications for our understanding of nerve injury–induced plasticity and pain.

## Abstract 2

A majority of small-diameter dorsal root ganglion neurons die following traumatic nerve injury. Discrete subpopulations are differentially lost, with Mrgprd-expressing nonpeptidergic nociceptors particularly vulnerable.

## 1. Introduction

Dorsal root ganglion (DRG) neurons represent a molecularly and functionally heterogeneous population. Under normal conditions, this diversity contributes to the ability of the somatosensory nervous system to detect a myriad of sensory stimuli that result in the perceptions of touch, temperature, itch, and pain. Following nerve injury, physiological changes in DRG neurons lead to hyperexcitability,^57^ which is a key pathological driver of neuropathic pain.^20,63^ Concomitant molecular changes in discrete subpopulations also occur, and these have recently been comprehensively described in single-cell^37,44^ and subpopulation-specific sequencing studies.^3^ These studies describe a transient and generalized reduction in the expression of subpopulation-specific genes following nerve injury.^3,37,44^

In addition to molecular changes, there is a rich literature describing the frank loss of DRG neurons following traumatic nerve injury in experimental rodent models.^24,50,53,56^ Some studies have suggested that neuron loss occurs in certain patient cohorts,^48,66^ but this is yet to be definitively demonstrated in humans. In rodents, most studies support a preferential loss of small cells that give rise to unmyelinated fibers^53^ but some contrasting studies describe the preferential loss of large cells^6^ or loss of cells of all sizes.^46^ Variation is evident across studies in terms of experimental species, age, type of injury, and quantification methods.^56^ Shi et al.^50^ used stereological counting methods to identify a 54% loss of DRG neuron number 4 weeks after “mid-thigh” sciatic nerve transection in C57BL/6 mice. Estimates for the degree of loss following commonly used nerve injury paradigms (eg, spared nerve injury [SNI] and sciatic nerve crush) are not available and because of the neurochemical changes following injury and the loss of subpopulation marker gene expression,^5,44,50^ the vulnerability of molecularly defined subpopulations has not been characterized. Moreover, more recent studies have cast doubt on the extent or even presence of DRG neuron death following nerve injury. One study which developed a deep learning approach to assess rat DRG cellular plasticity found no loss of neurons up to 2 weeks post-SNI,^49^ while another observed no loss of genetically labelled damaged DRG neurons 2 months after sciatic nerve crush.^44^

The issue of whether neuron loss occurs, and if so, in what subpopulations, is important. It will likely have implications for our understanding of reinnervation and functional recovery in patients. Furthermore, better insight will provide critical context for those investigating the plasticity that occurs following nerve injury and may inform therapeutic targeting of sensory neuron populations.

An expanding repertoire of transgenic recombinase driver lines now makes it possible to permanently label DRG neuron subpopulations and study their fate in rodent nerve injury paradigms. The aim of this study was to use this technology to characterize neuron loss after nerve injury and to test the hypothesis that loss is not equally distributed across molecular populations.

## 2. Methods

### 2.1. Animals

Mice were housed in groups in humidity- and temperature-controlled rooms with free access to food and water, on a 12-hour light–dark cycle, and with environmental enrichment. Animal procedures were performed under a UK Home Office Project Licence and in accordance with the UK Home Office (Scientific Procedures) Act (1986). All studies were approved by the Ethical Review Process Applications Panel of the University of Glasgow or Oxford and conform to the ARRIVE guidelines. Experiments were performed on adult male and female mice aged 7 to 16 weeks at the start of the experiments. All experimental cohorts contained a mix of male and female mice, apart from the cohort of Mrgprd^CreERT2^;Ai32 mice that underwent SNI_crush_ surgery, which was exclusively female. Details of transgenic lines are provided in Table 1. Tamoxifen was administered by i.p. injection of 20 mg/mL tamoxifen (Sigma-Aldrich) dissolved in wheat germ oil (doses described in Table 1). There were 2 instances where animals were excluded from data analysis: One (cyan fluorescent protein) Thy1-CFP died of unknown causes not related to the procedure and before the experimental endpoint, and one MrgD^CreERT2^;Ai32 exhibited no fluorophore expression and was therefore deemed to have been incorrectly genotyped. Group sizes were based on the extent of neuronal loss 28d following sciatic nerve transection identified by Shi et al.^50^ Given α = 0.05, power = 0.8, and an effect size of 4.81, power analysis projects that a group size of 3 mice would be needed.

**Table 1:** Transgenic lines used in the study.

Used name	Full name	Putative population	Ref	Source	Tamoxifen regime
Atf3^CreERT2^	Atf3^tm1.1(cre/ERT2)Msra^	Axotomised afferents	^13^	Gift: Dr Franziska Denk	50 mg/kg on days 0, 3, and 7 after surgery
Avil^FlpO^	Avil^tm1(flpo)Ddg^	Sensory neurons	^1^	Gift: Prof David Ginty	N.A.
MrgD^CreERT2^	Mrgprd^tm1.1(cre/ERT2)Wql^	Major class of nonpeptidergic neurons	^39^	The Jackson Laboratory (RRID:IMSR_JAX:031286)	General: 1x 50 mg/kg in adulthood, (>1 week before experiment) 3D volumetric analysis: 5x i.p. (0.5 mg/animal/day), beginning between P10 and P17
MrgD^ChR2-YFP^	Mrgprd^tm4.1(COP4)Mjz^	Major class of nonpeptidergic neurons	^59^	Mutant Mouse Resource & Research Centers (RRID:MMRRC_036112-UNC)	N.A.
Calca^CreERT2^	Calca^tm1.1(cre/ERT2)Ptch^	Peptidergic neurons	^51^	Gift: Prof Pao-Tien Chuang	1x 75 mg/kg in adulthood (>1 week before experiment)
Trpm8^FlpO^		Cold afferents	^4^	Gift: Dr Mark Hoon	N.A.
Thy1-CFP	B6.Cg-Tg(Thy1-CFP)23Jrs/J	Sample of myelinated afferents	^16^	The Jackson Laboratory (RRID:IMSR_JAX:003710)	N.A.
Th^CreERT2^	Th^tm1.1(cre/ERT2)Ddg^/J	C low threshold mechanoreceptors	^1^	Gift: Prof David Ginty; The Jackson Laboratory (RRID:IMSR_JAX:025614)	1x 50 mg/kg in adulthood (>2 weeks before experiment)
RC::FLTG	B6.Cg-*Gt(ROSA)26Sor*^*tm1.3(CAG-tdTomato,-EGFP)Pjen*^/J	Flp-mediated tdTomato; Cre+Flp-mediated GFP expression	^40^	The Jackson Laboratory (RRID:IMSR_JAX:026932)	N.A.
Ai14	B6.Cg-*Gt(ROSA)26Sor*^*tm14(CAG-tdTomato)Hze*^/J	Cre-mediated tdTomato expression	^33^	The Jackson Laboratory (RRID:IMSR_JAX:007914)	N.A.
Ai32	B6.Cg-*Gt(ROSA)26Sor*^tm32(CAG-COP4*H134R/EYFP)Hze^	Cre-mediated ChR2-eYFP expression	^32^	The Jackson Laboratory (RRID:IMSR_JAX:024109)	N.A.

CFP, cyan fluorescent protein; GFP, Green fluorescent protein; YFP, yellow fluorescent protein.

### 2.2. Spared nerve transection and crush surgeries

Spared nerve injury (transection of the common peroneal and tibial branches of the sciatic nerve; SNI_trans_) and common peroneal and tibial crush injury (SNI_crush_), in which nerve axons were severed but the epineurium remained intact, were performed as previously described.^12^ Anesthesia was induced with 3% to 5% isoflurane and then maintained at 1.5% to 2% as required. Analgesia, consisting of carprofen (10 mg/kg) and buprenorphine (0.05 mg/kg) (Glasgow) or carprofen (5 mg/kg) and local bupivacaine (2 mg/kg) (Oxford) was provided perioperatively. The left hindpaw was secured with tape in hip abduction, and the operative field (lateral surface of the thigh) was shaved. Ophthalmic ointment was applied to the eyes, and the shaved area was swabbed with chlorhexidine solution. A longitudinal incision was made in the skin at the lateral mid-thigh. Using blunt dissection, an opening was made through the biceps femoris, exposing the sciatic nerve and the 3 peripheral branches (sural, tibial, and common peroneal nerves). For SNI_trans_, the common peroneal and tibial nerves were ligated using a 6-0 Vicryl suture (Ethicon, Raritan, NJ), and a 1- to 2-mm piece distal to the suture was removed using spring scissors. For SNI_crush_, the exposed tibial and common peroneal nerves were clamped using a pair of fine hemostats (Fine Science Tools, Heidelberg, Germany) closed to their second clip, leaving the nerve branches intact but translucent. The muscle was closed with one 6-0 Vicryl suture (Ethicon), and the skin incision was closed with one 10 mm wound clip (Alzet, Cupertino, CA). Animals were monitored daily for self-mutilation, and no animals required sacrifice due to tissue damage.

### 2.3. FastBlue tracer injections

Mice were briefly anesthetized during the procedure, induced with 3% to 5% isoflurane, and then maintained at 1.5% to 2% as required. Hindlimbs were taped with the plantar surface of the paw facing up, and a custom, 26G removable needle with a 30° bevel, attached to a 25-µL Hamilton syringe, was inserted between the 2 distal-most footpads, towards the medial aspect of the hindpaw. The needle was then rotated 90°, so the bevel faced medially. Furthermore, 4-µL FastBlue (FB; 2% in sterile phosphate-buffered saline (PBS); CAS# 73819-41-7; Polysciences, Inc, Warrington, PA) per paw was then slowly injected, and the needle was left in place for 10 seconds, before rotating and carefully retracting to avoid backflow of FB along the needle track. This prevented the FB bolus from contacting the sural innervation territory of the lateral hindpaw, restricting it largely to the tibial innervation territory of the glabrous hindpaw skin.

### 2.4. Immunohistochemistry and image acquisition

Mice were anesthetized with an overdose of pentobarbital (20 mg) and transcardially perfused with a fixative containing 4% formaldehyde. L3 to L5 DRGs were removed and postfixed for another 2 hours, cryoprotected in 30% sucrose overnight, and then embedded in optimal cutting temperature media (OCT; Tissue Tek, Alphen aan den Rijn, the Netherlands). Dorsal root ganglia were sectioned on a Leica CM1950 cryostat at 30 µm, with every section collected serially on 5 Superfrost Plus slides (VWR, Lutterworth, United Kingdom) and each slide containing 1 in every 5 sections (4-7 sections per slide). One slide per DRG was selected at random and was washed with PBS, before being incubated with appropriate primary antibodies (Table 2) diluted in 5% normal donkey serum and 0.3% Triton X-100 in PBS for 3 days at 4°C. After PBS washes, slides were incubated with appropriate secondary antibodies (Table 2) in the same PBS/(normal donkey serum) NDS/Triton-X100 solution as for primaries, overnight at room temperature. Slides were washed and coverslipped with VectaShield Vibrance Hardset mounting media (Vector Labs, Newark, CA), with 4',6-diamidino-2-phenylindole included in mounting media where FB-labelled cells were not being examined. Sections were imaged using a Zeiss LSM900 Airyscan confocal microscope equipped with 405-, 488-, 561-, and 640-nm diode lasers. Full thickness, tiled, confocal image stacks with a 2- to 3-µm interval in the Z-axis were obtained through a 20× dry lens (0.8 NA) with the confocal aperture set to 1 Airy unit or less. All image capture was performed using Zen Blue Edition software (Carl Zeiss Microscopy GmbH, Jena, Germany), and analyses were performed using Zen Blue or FIJI.^45^

**Table 2:** Primary and secondary antibodies used in the study.

Antibody	Source	Identifiers	Working dilution
Anti-GFP (Chicken polyclonal)	Abcam, plc, Cambridge, United Kingdom	Cat#: ab13970 RRID: AB_300798	1:1000
Anti-NeuN (Guinea pig polyclonal)	Synaptic Systems, Göttingen, Germany	Cat#: 266004 RRID: AB_2619988	1:500
Anti-mCherry (Rat monoclonal)	Invitrogen, Waltham, MA; Thermo Fisher Scientific, United Kingdom	Cat#: M11217 RRID: AB_2536611	1:500
Anti-Atf3 (Rabbit polyclonal)	Novus Biologicals, Minneapolis, MN	Cat#: NBP1-85816 RRID: AB_11014863	1:500
Anti-NF200 (Rabbit polyclonal)	Sigma-Aldrich, Saint Louis, MO	Cat#: N4142 RRID: AB_477272	1:1000
Anti-TrkA (Goat polyclonal)	R&D Systems, Minneapolis, MN	Cat#: AF1056 RRID: AB_2283049	1:500
Anti-TDP43 (Rabbit polyclonal)	Abcam, plc, Cambridge, United Kingdom	Cat#: ab133547 RRID: AB_2920621	1:100
Anti-RFP (Mouse monoclonal)	Thermo Fisher Scientific, United Kingdom	Cat#: MA5-15257 RRID: AB_10999796	1:200
Anti-RFP (Chicken polyclonal)	Sigma-Aldrich, United Kingdom	Cat#: AB3528 RRID: AB_11212735	1:200
Alexa Fluor 488 Donkey Anti-Chicken IgY (Donkey polyclonal)	Jackson ImmunoResearch, Ely, United Kingdom	Cat#: 703-545-155 RRID: AB_2340375	1:500
Alexa Fluor 647 Donkey Anti-Guinea pig IgG (Donkey polyclonal)	Jackson ImmunoResearch, Ely, United Kingdom	Cat#: 706-605-148 RRID: AB_2340476	1:250
Rhodamine Red-X Donkey Anti-Rat IgG (Donkey polyclonal)	Jackson ImmunoResearch, Ely, United Kingdom	Cat#: 712-295-153 RRID: AB_2340676	1:100
Alexa Fluor 647 Donkey Anti-Rabbit IgG (Donkey polyclonal)	Jackson ImmunoResearch, Ely, United Kingdom	Cat#: 711-605-152 RRID: AB_2492288	1:250
Rhodamine Red-X Donkey Anti-Rabbit IgG (Donkey polyclonal)	Jackson ImmunoResearch, Ely, United Kingdom	Cat#: 711-295-152 RRID: AB_2340613	1:100
Alexa Fluor 546 Goat Anti-Chicken IgG (Goat polyclonal)	Thermo Fisher Scientific, United Kingdom	Cat#: A11040 RRID: AB_2534097	1:400
Alexa Fluor 488 Goat Anti-Rabbit IgG (Goat polyclonal)	Thermo Fisher Scientific, United Kingdom	Cat#: A11008 RRID: AB_143165	1:400
Alexa Fluor 546 Donkey Anti-Mouse IgG (Donkey polyclonal)	Thermo Fisher Scientific, United Kingdom	Cat#: A10036 RRID: AB_2534012	1:400

GFP, green fluorescent protein; RFP, red fluorescent protein

### 2.5. Image analysis

During all image quantification, the experimenter was blind to the experimental groups. For quantification of the total number of cells within the DRG, a modified optical dissector stereological method was used^11,18,47^ (Fig. S1, http://links.lww.com/PAIN/C84). To account for tissue shrinkage during processing, the mean thickness (*t*) of each section on one slide (ie, 1 in 5 sections) was calculated by taking the mean of the thickest and thinnest cell-containing regions (ie, not fiber tract-containing regions) of the section (NB: no optical correction to thickness was applied; given the use of a dry lens, this value will not reflect actual section thickness, though this was kept consistent throughout the study). The cell-containing, cross-sectional area (*a*) was then calculated, using the middle optical section from the series and drawing around the cell-containing regions. Section volume (*V*_*sec*_) was then calculated:

Vsec=t×a

Using the Cavalieri principle, the cell-containing volume of the DRG was calculated^11^:

$$VDRG=\bar{a}\times \bar{t}\times l$$

where $\bar{a}$ = mean cell-containing cross-sectional area, $\bar{t}$ = mean section thickness, and l = “length” of the DRG (determined from the total number of sections collected). The number of neurons per section (*N*_*sec*_) was quantified in all immunostained sections. This included only neurons with a visible nucleus (in the NeuN channel), excluded cells with a nucleus visible within the top frame of the Z-stack, and included any neurons with a nucleus visible in any other field within Z-stack, including the bottom frame of Z-stack. The cell density or the number of cells per unit vol (*N*_v_) was then calculated:

$$N_{v}=\frac{Nsec}{Vsec}$$

Finally, the total number of cells per DRG (*N*_*DRG*_) was calculated:

$$N_{DRG}=\bar{N_{v}}\times V_{DRG}$$

For quantification of the proportion of FB-labelled cells co-labelled with afferent subpopulation markers, initially, the total number of FB-filled neuronal cell profiles with a visible nucleus anywhere within the section was counted, with the observer blind to other channels. The other channel was then revealed, and instances of co-labelling were quantified. No stereological correction was applied, given that the similar size of neuronal nuclei would prevent over-counts of large neurons and that no comparisons of the total number of labelled cells were made. For soma area analyses, the area of neuronal soma expressing the appropriate marker was measured in the optical section within the Z-stack in which that neuron was at its largest, by drawing around the perimeter of the neuron in Fiji/ImageJ v2.14.0/1.54f.

### 2.6. Tissue clearing and 3D volumetric analyses

Dorsal root ganglia were extracted from animals 4 weeks post-SNI_trans_ for whole DRG analyses. In this study, tissue was extracted from a combination of MrgD^CreERT2^;Ai14, Th^CreERT2^;Ai14, and Calca^CreERT2^;Ai14 lines (mixed sex).^3^ One month after SNI_trans_, animals were transcardially perfused with sterile saline followed by a fixative containing 4% formaldehyde. Ipsilateral and contralateral L4 DRG were removed and postfixed for 24 hours on a shaker at room temperature before being washed in PBS and stored at −80°C in CI-VM1 (35% dimethyl sulfoxide, 35% ethylene glycol in PBS) until clearing. Tissue clearing was then performed as previously described.^67^ In brief, the tissue was exposed to a gradient of 1-propanol containing 0.3% triethylamine (30, 50, 75, 90, 95, 100, 100%) and washed in this solution at 37°C for 24 hours. The tissue was then rehydrated in PBS and labelled with primary antibodies for 1 week at 37°C (mouse anti-TDP43 and 2x anti-RFP, Table 2). The tissue was washed for 24 hours and incubated with appropriate secondary antibodies (Table 2) for another week at 37°C. The tissue was subsequently washed for 24 hours, dehydrated again in increasing concentrations of 1-propanol containing 0.3% triethylamine, and mounted in benzyl alcohol with benzyl benzoate (1:2 ratio) containing 0.3% triethylamine on glass slides with silicone spacers. Imaging was performed on an Olympus spinning disk confocal microscope at 20x, with 2-µm z-steps. The tissue was stored at 4°C for ∼16 months before imaging, so only the tissue that remained transparent at this time was used for downstream analyses. Volumetric analyses were performed using Imaris using the “spots” feature with region growth (to allow for different-sized spots), background subtraction, and point spread function elongation (standard 2 × XY). Initial spot diameters were set based on MrgD^CreERT2^;Ai14 nuclear size (as labelled by red fluorescent protein (RFP)). Spot classification was then performed blind by adjusting the quality threshold to balance detection in superficial and deep tissue. This step was necessary due to differences in tissue quality after long-term storage. Any labelled spots in the adjacent nerve were then deleted (eg, labelled Schwann cells or debris). Count and volumetric data were then exported for analysis in R. Data were filtered for very small (<5 µm^3^) and very large (>2000 µm^3^) spots to further remove any debris, labelled satellite glia or doublets within the ganglia. In both cases, these filters were approximate and did not exclude the possibility that some spots correspond to either class in the final dataset. The upper limit of the “small” DRG nuclei size category was defined as the upper bound of 32 easily identifiable MrgD+ nuclei (258 µm^3^). The boundary between “medium” and “large” bins (400 µm^3^) was less clearly defined in the samples and was therefore set as the approximate midpoint of the volume distribution. A combined size category for all nuclei greater than 258 µm^3^ was also examined, and the results mirrored those of “medium” and “large” bins.

### 2.7. Gene Ontology

Gene Ontology term analyses were performed on previously published mouse subtype RNA-seq after SNI (GSE216444^3^). In this study, subtype-specific bulk RNA-seq was performed on 5 transgenic mouse lines through reporter labelling and fluorescence activated cell sorting. spliced transcripts alignment to a reference was used to map reads to the GRCm38 (mm10) Mouse Genome,^14^ and Samtools was used to sort, index, and merge Binary Alignment Map files in line with published reports.^28^ Quality control was performed as per Barry et al.^3^ Downstream analyses were performed using DESeq2 on grouped male and female samples.^31^ For differentially expressed genes (false discovery rate) (FDR < 0.05, LFC >1) (log-fold change), GO analyses were performed using the Wallenius method using goSeq (R). In this study, significantly regulated terms related to cell death and apoptosis with more than 10 genes were examined. Filtered count data of expressed and nondifferentially expressed genes were used as a background.

### 2.8. Dorsal root ganglion culture

Dorsal root ganglia were dissected from MrgD^CreERT2^;Ai32 and Calca^CreERT2^;Ai32 mice >1 week after dosing with tamoxifen and enzymatically digested at 37°°C for 80 minutes in dispase type II (4.7 mg/mL) plus collagenase type II (4 mg/mL) (Worthington Biochemical), as described previously.^63^ Mechanically dissociated cells were plated onto laminin/poly-D-lysine (R&D Systems, Minneapolis, MN) treated coverslips in complete Neurobasal Plus medium (Neurobasal Plus media supplemented with 2% (vol/vol) B27 Plus, 1% N2, 1% Glutamax, and 1% antibiotic–antimycotic [ThermoFisher Scientific, Waltham, MA]). Mouse nerve growth factor (GF) (50 ng/mL; nerve growth factor (NGF), PeproTech, Cranbury, NJ) and 10 ng/mL glial-derived neurotrophic factor (GDNF, PeproTech) were added to the media under some conditions. Cytosine β-D-arabinofuranoside (4 µM) was added to the media for 24 hours the day after plating to reduce the proliferation of nonneuronal cells. Media was refreshed 3 times per week thereafter. Cultures were fixed for 10 minutes at room temperature with 4% paraformaldehyde and subsequently processed by immunocytochemistry (described earlier).

### 2.9. Statistical analysis

Data are expressed as mean ± SEM unless otherwise specified, and *P* values of less than 0.05 were considered significant. Power calculations were performed using G*Power 3.1.9.7.^15^ A quantitative Venn diagram was created using BioVenn.^25^ All other statistical analyses were performed in Prism 10 (GraphPad Software, Inc, Boston, MA) or R using paired *t* tests or 1- or 2-way RM ANOVAs (repeated measures analysis of variance), where appropriate. Normality was assessed by the Shapiro–Wilk test. If the main analysis of variance effect was significant, Šídák or Tukey multiple comparisons tests were performed. To compare population distributions of soma cross-sectional area or volume, Kolmogorov–Smirnov tests were performed.

## 3. Results

### 3.1. Peripheral nerve injury induces a loss of small neurons from the dorsal root ganglion

**Figure 1.**
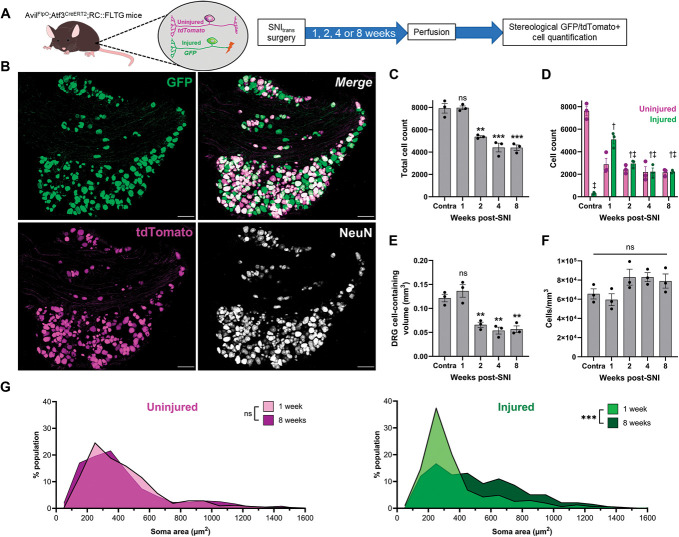
SNI_trans_ induces death of small primary afferent neurons, accompanied by a reduction in volume, not cell density, of the dorsal root ganglion. (A) Approach to differentially labelled intact afferents with tdTomato and damaged afferents with GFP after peripheral nerve injury using the Avil^FlpO^;Atf3^CreERT2^;RC::FLTG mouse line and schematic of experimental timeline. (B) Representative image of GFP, tdTomato, and NeuN expression in an L4 DRG, 2 weeks after SNI_trans_. Scale bars = 100 µm. (C and D) Stereological quantification of the total number of DRG neurons (C) or number of axotomized and intact neurons (D) in the L4 DRG 1, 2, 4, and 8 weeks after SNI_trans_ or contralateral (contra) to injury. (C) One-way ANOVA with Tukey posttests; *F*_4,10_ = 37.98, *P* < 0.001. (D) Two-way RM ANOVA; Timepoint × Color interaction *F*_4,10_ = 39.04, *P* < 0.001, n = 3 mice; Tukey posttests (between injured groups): †*P* < 0.05 vs contra, ‡*P* < 0.05 vs 1-week. (E) Volume of DRG-containing cells (ie, excluding white matter tracts) following SNI_trans_. One-way ANOVA with Tukey posttests; *F*_4,10_ = 21.25, *P* < 0.001, n = 3. (F) Neuronal density within the DRG following SNI_trans_. One-way ANOVA; *F*_4,10_ = 2.77, *P* = 0.09, n = 3. (G) Population distribution of uninjured and injured afferents by cross-sectional area, 1 and 8 weeks post-SNI_trans_. Kolmogorov–Smirnov tests of cumulative distributions; Uninjured: D = 0.08, *P* = 0.18; Injured: D = 0.32, *P* < 0.001; n = 310 to 427 neurons from 3 mice. *P < 0.05, **P < 0.01, ***P < 0.001 vs contra. ANOVA, analysis of variance; DRG, dorsal root ganglion; GFP, green fluorescent protein.

To assess the gross loss of neurons from DRG following nerve injury, we generated the Avil^FlpO^;Atf3^CreERT2^;RC::FLTG mouse line in which naïve and axotomized sensory neurons were differentially labelled. In this mouse line, all neurons express tdTomato (Flp-dependent) in the naïve state and switch to expressing green fluorescent protein (GFP) upon axonal damage and concurrent tamoxifen treatment (Flp- and Cre-dependent) (Figs. 1A and B). Following pilot experiments to optimize tamoxifen dosing regimen, this approach was both highly efficient and specific (with the caveat that it was necessary to wait for several days after nerve injury for Cre-induced GFP expression): 14 days after SNI_trans_ surgery, GFP was expressed by 99.1 ± 0.6% of Atf3-expressing ipsilateral L4 DRG neurons, while we observed GFP in only 4.6 ± 0.7% of contralateral DRG neurons (Figs. S2A–D, http://links.lww.com/PAIN/C84). We then used a stereological approach to quantify the total number of neurons in L4 DRG ipsilateral to injury 1, 2, 4, and 8 weeks after SNI_trans,_ as well as contralateral to injury. One week after SNI_trans_, we observed 7809 ± 153 neurons per DRG; this was not significantly different to the number of neurons in the contralateral DRG (7917 ± 349), whereas cell number approximately halved by 8 weeks postinjury to 3963 ± 410 neurons per DRG (Fig. 1C). Separating analysis into intact vs axotomized afferents revealed that only axotomized afferents were lost, with no difference observed in numbers of intact afferents (Fig. 1D). Between 1 and 8 weeks after injury, we observed a 61.0 ± 7.0% decrease in the number of GFP+ neurons. This loss of injured afferents resulted in a loss of neuron-containing (ie, excluding white matter regions) DRG volume (Fig. 1E), but not neuron density (Fig. 1F). Cell loss predominantly occurred between 1 and 2 weeks postinjury and stabilized after this timepoint. Population distributions of the cross-sectional area of nucleated, tdTomato-expressing cell profiles were not significantly different at 1 vs 8 weeks post-SNI_trans_, in contrast to GFP-expressing/injured afferents, in which a loss of a population of small afferents at 8 weeks postinjury was observed (Fig. 1G).

**Figure 2.**
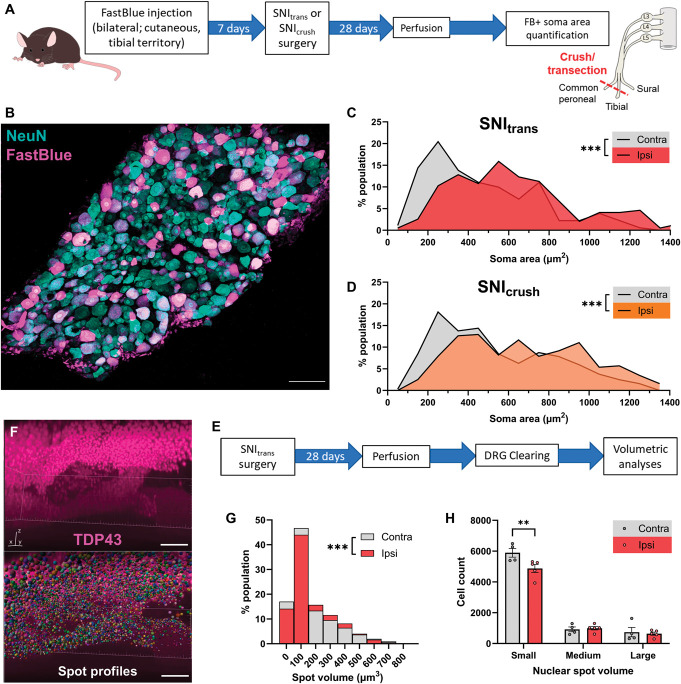
Spared nerve crush and transection lead to a loss of small DRG neurons. (A) Approach to restrict analysis to damaged afferents: a subcutaneous injection of the tracer FB into both hindpaws labelled tibial afferents, before unilateral SNI_trans_ or SNI_crush_ surgery. (B) Representative image of FB labelling and NeuN immunostaining in the L4 DRG. The image is a projection of optical sections at 3-µm intervals through the entirety of a 30-µm-thick tissue section. Scale bar = 100 µm. (C and D) Quantification of the cross-sectional area of FastBlue labelled DRG neurons ipsilateral and contralateral to SNI_trans_ (C) or SNI_crush_ injury (D) reveals a loss of small afferents and subsequent shift in population distribution. Kolmogorov–Smirnov tests of cumulative distributions; SNI_trans_: D = 0.25, *P* < 0.001; n = 183 or 191 neurons from 3 mice; SNI_crush_: D = 0.22, *P* < 0.001, n = 319 or 325 neurons from 3 mice. (E) Experimental approach for whole DRG volumetric analyses after SNI_trans_. (F) Representative 3D rendering of TDP-43 profiles and corresponding nuclear spot profiles following Imaris-based spot detection feature. Scale bar = 100 µm. (G) Quantification of DRG nuclear spot volume ipsilateral and contralateral to SNI_trans_. Kolmogorov–Smirnov tests of cumulative distribution: D = 0.06, *P* < 0.001, n = 30,206 (contra) or 32,544 (ipsi) nuclei from 4 (contra) or 5 (ipsi) mice. (H) Total number of nuclear spots, by size, per DRG. Two-way RM ANOVA; size bin × injury interaction: *F*_2,14_= 8.26, *P* = 0.004; n = 4 to 5 mice; Šídák multiple comparisons tests: **P < 0.01. ANOVA, analysis of variance; DRG, dorsal root ganglion; FB, FastBlue; RM, repeated measures.

SNI_trans_ resulted in a mixed population of axotomized and intact afferents within the L4 DRG. Therefore, we developed an approach to restrict our analysis to axotomized afferents, without relying on transgenic labelling, and used this as a complementary approach to confirm our findings. We injected the neuronal tracer FB into the glabrous, tibial innervation territory of both hindpaws 1 week before common peroneal and tibial transection (SNI_trans_) or crush (SNI_crush_) surgeries (Figs. 2A and B). FastBlue-uptake was complete across neurons of all sizes by 1 week (Fig. S3, http://links.lww.com/PAIN/C84), so this approach allowed us to profile a sample of the axotomized afferents. Both SNI_trans_ (Fig. 2C) and SNI_crush_ (Fig. 2D) injuries resulted in a rightward shift in population distributions of the cross-sectional area of nucleated, FB-labelled DRG neurons when compared with contralateral DRG, consistent with a loss of small afferents post–nerve injury.

As a third complementary approach, we applied semiautomated volumetric analyses of nuclei size following tissue clearing. In this study, whole DRGs were cleared 4 weeks after SNI_trans_ for nuclei counting in “complete” tissue (Figs. 2E–H). Nuclei were labelled by TDP-43, in line with the study by West et al.,^67^ and were quantified using Imaris software (Fig. 2F, Video 1). We observed a slight but significant rightward shift in nuclear spot volume population distribution 4 weeks after SNI_trans_ (Fig. 2G). In addition, there was a significant reduction in the number of small but not medium or large nuclear spots, in support of a loss of small-diameter neuron populations (Fig. 2H).

**Video 1:** Whole DRG TDP-43 expression and nuclear spot profiles. Representative 3D rendering of TDP-43 profiles and corresponding nuclear spot profiles following Imaris-based spot detection feature.

Together, our data derived from several different experimental approaches show that a population of small-diameter afferents are lost following peripheral nerve injury.

### 3.2. Spared nerve crush or transection results in death of Mrgprd-expressing neurons

**Figure 3.**
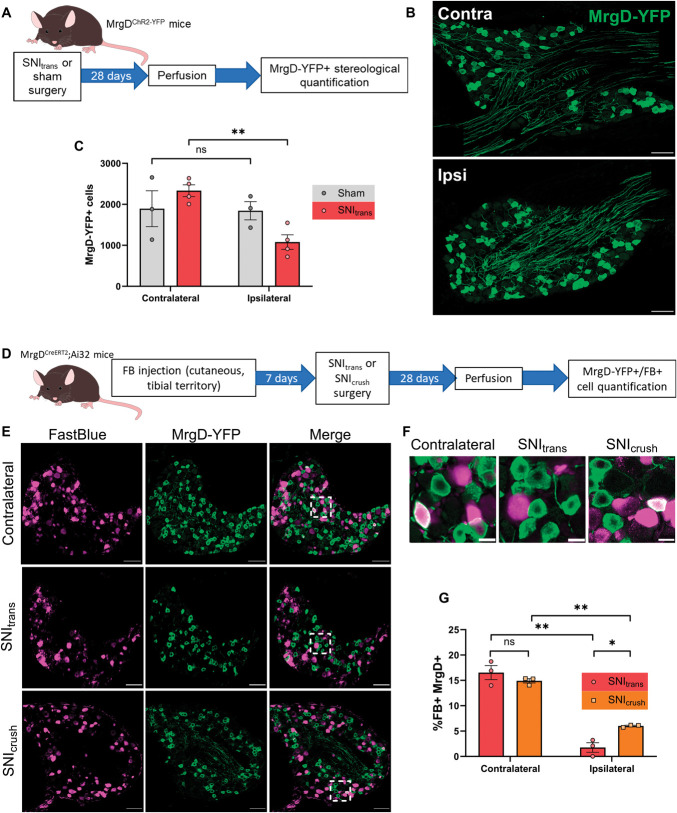
Spared nerve crush or transection results in death of nonpeptidergic neurons. (A) Schematic of experimental approach for (B and C). (B) MrgD^ChR2-YFP^ L4 DRGs 4 weeks after SNI, contralateral or ipsilateral to injury. Images are projections of optical sections at 3-µm intervals through the entirety of 30-µm-thick tissue sections. Scale bars = 100 µm. (C) Quantification of total number of MrgD-YFP+ cells per L4 DRG 4 weeks after SNI revealed a significant loss in ipsilateral DRG. Two-way RM ANOVA with Šídák multiple comparisons tests; Side x Treatment interaction: F_1,5_ = 9.23, *P* = 0.029; n = 3 mice. (D) The experimental approach used to generate data presented in (E–G). (E and F) MrgD-YFP expression and FB labelling in the L4 DRG, 14 days after SNI or crush surgery or contralateral to injury. White boxes represent regions enlarged in (F). Scale bars = 100 µm (E) or 20 µm (F). (G) The proportion of FB-labelled DRG neurons decreased after spared nerve crush injury, and co-labelling is almost completely absent after SNI. Two-way RM ANOVA with Šídák multiple comparisons tests; side × injury interaction: *F*_1,4_ = 7.80, *P* = 0.049; n = 3 mice. Posttests: *P < 0.05, **P < 0.01. ANOVA, analysis of variance; DRG, dorsal root ganglion; SNI, spared nerve injury; FB, FastBlue; RM, repeated measures.

To date, determining cell loss among specific populations of afferent neurons has proved challenging due to the downregulation of subpopulation-specific marker genes following axonal transection.^37,44^ To overcome this issue, we took advantage of transgenic strategies to label populations in a manner that persisted after injury. Owing to the bias for the loss of small neurons and the known loss of IB4-binding central terminals postinjury,^36^ we initially focused on nonpeptidergic nociceptive neurons. We used MrgD^ChR2-YFP^ mice to identify neurons belonging to the largest of the 3 classes of nonpeptidergic nociceptors, NP1.^55,59^ To determine whether these neurons are lost following nerve injury, we used a stereological method to quantify L4 DRG MrgD-YFP^+^ (yellow fluorescent protein) neurons 28 days after sham surgery or SNI_trans_ (Figs. 3A and B). SNI_trans_, but not sham, resulted in a significant decrease (54.0 ± 6.6%) in the total number of MrgD-YFP^+^ neurons in L4 DRG (Fig. 3C).

Yellow fluorescent protein expression in MrgD^ChR2-YFP^ mice is driven by the endogenous *Mrgprd* promotor, which has been reported to be upregulated or downregulated following axonal damage.^44,58^ Such changes in promoter activity could affect the proportion of nonpeptidergic nociceptors identified by YFP expression. Therefore, to verify these findings, we used MrgD^CreERT2^;Ai32 mice and tamoxifen administration before injury, to permanently label *Mrgprd-*expressing afferents with ChR2-YFP (Figs. 3D–F). We then tested whether the proportion of cutaneous tibial afferents that were YFP^+^ was altered following nerve injury. Following hindpaw FB injection, ∼15% of contralateral, FB-labelled DRG neurons expressed YFP. This was reduced to 6.0 ± 1.2% 28 days after SNI_crush_ injury and to only 1.7 ± 0.9% 28 days after SNI_trans_ (Fig. 3G). Uptake by uninjured YFP^+^ neurons was equivalent 7 and 35 days after FB injection, demonstrating that this reduction was not because 7 days were insufficient for YFP^+^ neurons to fully uptake FB (Fig. S3C, http://links.lww.com/PAIN/C84). No significant difference in the percentage of FB-labelled YFP^+^ DRG neurons between ipsilateral and contralateral DRG was observed at 7 days following SNI_trans_ (Figs. S4A and B, http://links.lww.com/PAIN/C84), demonstrating that loss occurred after this timepoint. Analysis of the cross-sectional soma area of FB-labelled, YFP^+^ neurons in uninjured DRG revealed an area of 361 ± 138 µm^2^ (mean ± SD) (Fig. S4C, http://links.lww.com/PAIN/C84), which is a distribution profile matching those neurons presumed lost. Collectively, these data show that peripheral nerve injury results in a substantial loss of nonpeptidergic, *Mrgprd*-expressing neurons, with SNI_trans_ (ie, an unrepaired axonal transection) resulting in an almost complete loss of this population.

### 3.3. Spared nerve injury induces a loss of Trpm8^+^ and calcitonin gene-related peptide^+^ but not myelinated dorsal root ganglion neurons

**Figure 4.**
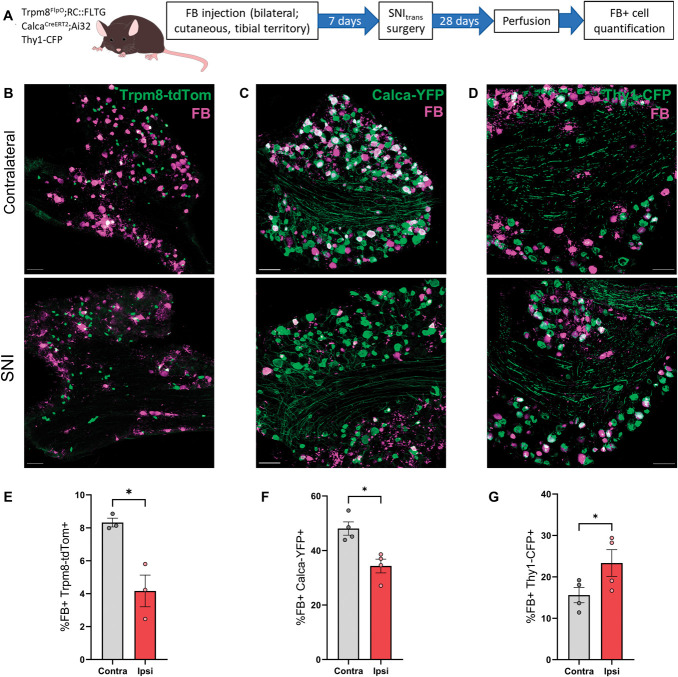
Spared nerve injury induces a loss of Trpm8+ and CGRP+ but not myelinated DRG neurons. (A) Schematic of experimental approach. (B–D) FastBlue labelling and Trpm8-tdTom (B), Calca-YFP (C), or Thy1-CFP expression (D) 28 days after SNI_trans_ in the L4 DRG, contralateral (top) or ipsilateral (bottom) to injury. Images are projections of optical sections at 3-µm intervals through the entirety of 30-µm-thick tissue sections. Scale bars = 100 µm. (E–G) Quantification of the proportion of FB-labelled neurons also expressing Trpm8-tdTom (E), Calca-YFP (F), or Thy1-CFP (G) in L4 DRG contralateral or ipsilateral to SNI_trans_. Paired *t* tests; Trpm8-tdTom: t_2_ = 5.31, *P* = 0.034, n = 3 mice; Calca-YFP: t_3_ = 4.12, *P* = 0.026, n = 4 mice; Thy1-CFP: t_3_ = 4.42, *P* = 0.022, n = 4 mice. *P < 0.05. CFP, cyan fluorescent protein; CGRP, calcitonin gene-related peptide; DRG, dorsal root ganglion; FB, FastBlue.

Loss restricted to nonpeptidergic nociceptors would not fully account for the degree of total neuron loss that we observed. Therefore, we studied a range of other subpopulations, both small and large in diameter, for their vulnerability to injury-induced loss. To investigate potential loss of Trpm8^+^ (cold-sensitive), calcitonin gene-related peptide^+^ (CGRP) (peptidergic), and myelinated subpopulations of DRG neurons following nerve injury, we applied our FB-labelling approach in Trpm8^FlpO^;RC::FLTG (FlpO-dependent tdTom expression), Calca^CreERT2^;Ai32 (Cre-dependent ChR2-YFP expression) and Thy1-CFP mice, respectively (Figs. 4A–D). Trpm8-tdTom was expressed by a population of small-diameter, putative cold-sensitive neurons (Fig. 4B), accounting for 8.3 ± 0.27% of FB-labelled neurons in contralateral DRG. This decreased to 4.2 ± 0.96% ipsilateral to SNI_trans_ injury (Fig. 4E), indicating a partial loss of Trpm8^+^ afferents. When examining peptidergic afferents, we found that 48.1 ± 2.42% of FB-labelled neurons in contralateral DRG were Calca-YFP^+^, compared with 34.3 ± 2.54% 4 weeks after SNI_trans_ injury (Figs. 4C and F), consistent with a partial loss of CGRP^+^ afferents. We used a Thy1-CFP line that demonstrates consistent expression postinjury^61^ and labels a sample of medium/large diameter myelinated afferents. CFP was largely restricted to NF200^+^ neurons, labelling 56% of this population. Expression was present in a heterogenous population of nociceptive (TrkA+) and nonnociceptive (TrkA-) myelinated neurons (Fig. S5, http://links.lww.com/PAIN/C84). Contralateral to injury, 15.6 ± 1.8% of FB-labelled neurons expressed Thy1-CFP (Figs. 4D and G). In contrast to unmyelinated subpopulations, this proportion was higher in ipsilateral DRG following SNI_trans_ (23.3 ± 3.2%), consistent with no (or minimal) loss of Thy1-CFP-expressing afferents, accompanied by a loss of Thy1-CFP-negative neurons. We did not observe significant alterations in the population distributions of the cross-sectional area of surviving, damaged Trpm8-tdTom^+^, Calca-YFP^+^, or Thy1-CFP^+^ DRG neurons when compared with DRG contralateral to injury (Fig. S6A–C, http://links.lww.com/PAIN/C84), indicating that any loss of neurons within specific neuronal subpopulations was not biased towards soma size. Collectively, these data show that unrepaired axonal damage to peripheral sensory neurons induces a partial loss of Trpm8^+^ and CGRP^+^ subpopulations, but no major loss of myelinated afferents.

Based on our findings of preferential loss of nonpeptidergic nociceptors, we re-analyzed a previous population-specific transcriptomic dataset of mouse DRG neurons following nerve injury for potential upregulation of cell death pathways (Fig. S7, http://links.lww.com/PAIN/C84).^3^ We found that early after injury (3 days post-SNI_trans_), nonpeptidergic (MrgD^CreERT2^-expressing) neurons showed enhanced enrichment of GO terms associated with apoptosis, in contrast to a broad population of nociceptors (labelled with Scn10a^CreERT2^), peptidergic nociceptors (Calca^CreERT2^), C-LTMRs (Th^CreERT2^), and Aβ-RA (rapidly adapting) and Aδ-LTMRs (Aδ/Aβ-LTMR, Ntrk2^CreERT2^;Advillin^FlpO^), in which there was less or no enrichment of cell death pathways. By 4 weeks, only C-LTMR and Aδ/Aβ-LTMR subtypes show any overrepresentation of cell death pathways (in the populations studied). Both injury-specific and apoptotic signatures in nonpeptidergic neurons were no longer significantly enriched, consistent with a loss of axotomized nonpeptidergic afferents by this late timepoint postinjury. These data suggest that apoptotic pathways are upregulated acutely after injury in a cell-type-specific manner.

### 3.4. Mrgprd dorsal root ganglion neurons are sensitive to loss in vitro

**Figure 5.**
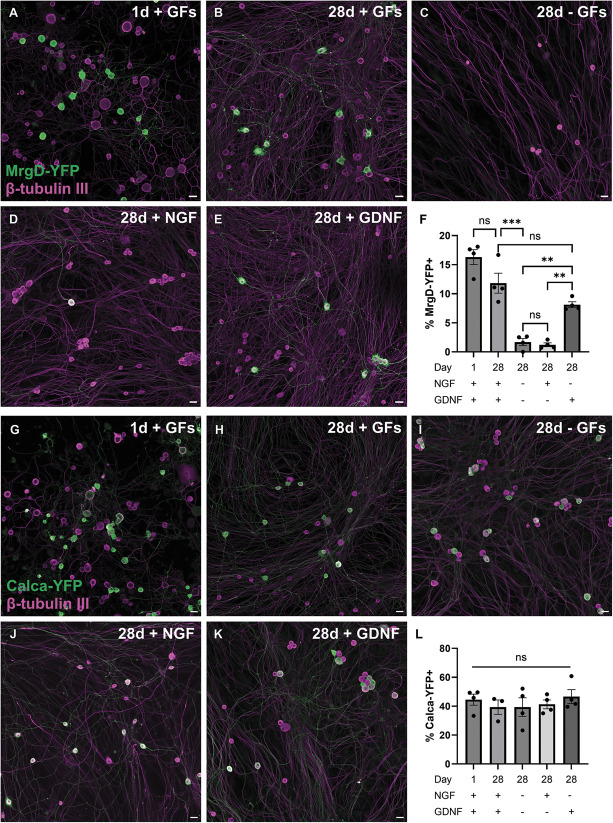
Neurotrophic support ameliorates MrgD+ cell loss in vitro. (A–E) Representative fields of view of MrgD-YFP and β-tubulin III expression in neuronal cultures of isolated MrgD^CreERT2^;Ai32 DRGs, 24 hours (A) or 4 weeks (B–E) after plating, with the addition of the growth factors (GFs) NGF, GDNF, both, or neither. Scale bars = 20 µm. (F) Quantification of the percentage of neurons that express MrgD-YFP under the conditions shown in (A–E). One-way ANOVA with Tukey posttests; *F*_4_,_15_ = 39.7, *P* < 0.001; n = 4 mice. Posttests: *P < 0.05, **P < 0.01, ***P < 0.001. (G–K) Representative fields of view of Calca-YFP and β-tubulin III expression in neuronal cultures of isolated Calca^CreERT2^;Ai32 DRGs, with the addition of NGF, GDNF, both, or neither. Scale bars = 20 µm. (L) Quantification of the percentage of neurons that express Calca-YFP. One-way ANOVA; *F*_4_,_14_ = 0.46, *P* = 0.76; n = 3 to 4 mice. ANOVA, analysis of variance; DRG, dorsal root ganglion; GDNF, glial-derived neurotrophic factor; NGF, nerve growth factor; YFP, Yellow fluorescent protein.

Earlier studies postulated that a lack of neurotrophic support underlies neuronal loss, which is supported by the observation that exogenous GDNF treatment at the time of injury, or shortly after, rescues the loss of IB4-binding central terminals posttransection.^5^ We sought to use the DRG neurons from MrgD^CreERT2^;Ai32 mice to test this postulate and establish an in vitro platform capable of probing the molecular basis of loss, with axonal transection during isolation providing a correlate for in vivo nerve injury (Figs. 5A–E). Twenty-four hours after plating, YFP was expressed by 16.3 ± 1.3% of DRG neurons, which was reduced to 11.8 ± 1.7% after 28 days of culture in the presence of exogenous GFs, NGF and GDNF (Fig. 5F). However, in the absence of GFs, YFP^+^ neurons only accounted for 1.7 ± 0.6% of neurons after 28 days, accompanied by an apparent reduction in the overall number of neurons within the culture, despite all conditions being seeded at the same initial density (Figs. 5C and F). YFP^+^ cell loss was partially rescued by the presence of GDNF, but not NGF alone, in the culture media (Figs. 5D–F). These results contrasted with experiments using neurons derived from Calca^CreERT2^;Ai32 mice, in which we observed no change in the proportion of neurons that were Calca-YFP^+^ after 28 days in culture, regardless of exogenous GF addition (Figs. 5G–L). Collectively, these data support the use of DRG cultures to probe the mechanisms underlying selective loss of sensory neurons following nerve injury and suggest a role for trophic support, particularly by GDNF signaling, in preventing the loss of nonpeptidergic nociceptors.

## 4. Discussion

We present data herein to support the hypothesis that traumatic nerve injury in rodents leads to a profound loss of small-diameter DRG neurons. Taking advantage of newly developed transgenic recombinase driver lines, we have shown that loss is biased across molecularly defined subpopulations. Nonpeptidergic nociceptive neurons are particularly susceptible to loss, with almost all Mrgprd^+^ axotomized afferents lost following an unrepaired transection injury (SNI_trans_) and roughly half lost following a model which contrastingly allows for nerve regenerations (SNI_crush_). Finally, we have observed that the vulnerability of Mrgprd^+^ neurons extends to the in vitro setting and provide data to support the hypothesis that loss is driven by a lack of neurotrophic support following injury.

### 4.1. Neuronal loss

The question of whether DRG neurons die following traumatic injury has been addressed by several groups over the last few decades. Despite contrasting findings on the extent, timing, and form that loss takes, most studies have observed frank loss of DRG neurons.^6,38,46,53^ However, more recent studies using recombinase driver lines and novel machine-learning approaches have cast doubt on this consensus.^44,49^ Our data strongly support the loss hypothesis and suggest that approximately 60% of axotomized afferents die within 2 weeks of SNI. The discrepancy between our findings and other recent studies may be partly explained by the sampling method used to estimate neuronal numbers. For example, Schulte et al.^49^ developed a novel machine-learning approach and found no reduction in neuron density across serial sections of rat DRG following SNI, and they inferred from this that frank loss did not occur. Our results are congruous, in that we also observed no reduction in neuron density. However, we found a substantial loss in the total neuron-containing volume of injured DRG, which underlies our contrasting conclusion of frank loss. Of note, morphological volumetric analysis and MRI have also previously demonstrated volume loss in both rodent and human DRG following nerve injury.^35,65,66^ These findings occur despite a major increase of nonneuronal cells in the injured DRG^30^ and support the notion that the total DRG neuron number is decreased.

### 4.2. Selectivity of neuron loss

While definitively characterizing loss of molecularly defined subpopulations was challenging before the advent of recombinase driver lines, a consensus emerged that small-diameter neurons are more vulnerable to nerve injury–induced loss.^50,53^ Our data support this consensus and extend it to reveal that while there is a generalized partial loss of C-fiber populations including CGRP- and Trpm8-expressing neurons, Mrgprd-expressing neurons are particularly sensitive to loss. This selective vulnerability has been hinted at previously by the stark reduction in the number of DRG neurons and their central terminals that bind IB4 and express canonical markers such as the P2X_3_ receptor following nerve injury.^5,8,29,36^ Type 1a glomeruli are also reduced in lamina II, suggesting a structural loss of central terminals and not simply a loss of IB4-binding.^2^ However, it was not clear whether these data represented phenotypic changes in nonpeptidergic nociceptors or frank loss of neurons. We describe neuron loss that is delayed (occurring >7 days postinjury) with respect to histochemical and structural changes (occurring 1-5 days postinjury^2,29^), suggesting that these changes precede and are not in themselves indicative of neuron loss.

The vulnerability of Mrgprd-expressing neurons is congruous with recent subpopulation bulk RNA-seq data, which found that SNI-related gene expression signatures were less evident in Mrgprd-expressing and C-LTMR neurons at later timepoints, compared with other populations in injured DRG.^3^ This could be explained by a loss of axotomized neurons of these classes and therefore sampling of only uninjured neurons at this timepoint.^24,43,64^ In terms of the transcriptional response to injury, nonpeptidergic nociceptors show enrichment of individual proapoptotic factors early after injury,^23,68^ and we extend these results in this study, by describing a subpopulation-specific enrichment of GO terms associated with apoptosis that is evident as early as 3 days after injury. Such data and single-cell transcriptomic profiling of all DRG neurons following injury^37,44^ may offer the opportunity to elucidate the cell death pathways engaged and upstream effectors that enrich this process to nonpeptidergic nociceptive neurons.

### 4.3. Implications for pain pathogenesis

Neuronal loss has been proposed as a key contributor to poor functional recovery following nerve injury,^54^ and biased survival of different afferent types might be expected to contribute to modality-specific sensory deficits. Beyond loss of function, does DRG neuron loss contribute to chronic pain, in either an adaptive or maladaptive manner? Intrathecal delivery of GDNF is neuroprotective and reverses the reduction in the number of IB4-binding DRG neurons and central terminals seen following transection.^5^ Treatment is concurrently analgesic and abrogates pain-related behaviors.^7,60^ However, the pleiotropic nature of GDNF makes it impossible to directly attribute the analgesic effects to the reversal of neuron loss. Indeed, it is possible that GDNF exerts its effect by actions on intact nonpeptidergic nociceptive afferents,^52^ activation of which is known to drive aversive behaviors in the neuropathic state.^62^ These data leave the contribution of nonpeptidergic nociceptor loss to behavior in the GDNF treatment paradigm ambiguous. Other pharmacological approaches have been found effective at reversing a neuronal loss in rodent models, but the impact on pain behavior was not studied.^21,22^

Rodents develop marked mechanical and thermal hypersensitivity rapidly following nerve injury and before timepoints at which neuron loss is observed.^10^ This lack of a temporal correlation may suggest a limited contribution to evoked hypersensitivities. The temporal profile of ongoing tonic pain (eg, pain aversiveness as measured by condition place preference assays^26^) is less defined and so is its correlation to the timing of neuron loss.

There are many anatomical sites within the somatosensory nervous system where differential loss of sensory neuron populations could impact neurobiology. For example, loss of cutaneous afferents may afford more opportunity for plasticity in reinnervation patterns, such as collateral sprouting of uninjured or surviving afferents, and the types of nerve endings made by different molecular subpopulations.^17,27^ It also seems likely that the death of many neurons within a DRG could contribute to the expansion and activation of immune cell types, which are known to play a major role in neuropathic pain.^30,69^ Finally, under normal conditions, peripheral sensory input is integrated into the dorsal horn of the spinal cord by complex interneuron circuitry. Many spinal circuits are engaged by convergent input from different afferent types.^9,41,70^ Therefore, selective loss of input from discrete afferent types could undoubtedly impact the normal processing of remaining afferent signals.^34^ Experimentally abrogating neuronal loss may be a fruitful approach to assess the contribution to nervous system plasticity (adaptive or maladaptive) following injury. In this regard, our in vitro readout would be a useful experimental platform to help delineate the precise cell death pathways and signaling cascades engaged (which could then be experimentally manipulated). Such studies should consider that plasticity may evolve over time. The loss of IB4^+^ central terminals is transient following crush and has even been observed to reverse at longer timepoints following SNI_trans_.^36^ These observations, in conjunction with ours of loss of neurons, raise the intriguing question of the source of such central reinnervation.

### 4.4. Study limitations

Our efforts focused on traumatic nerve injury paradigms owing to previous contrasting results using these robust and reproducible experimental models. We did not extend our studies to systemic neuropathy models, such as chemotherapy or diabetic neuropathy. A recent postmortem analysis reported a neuronal loss in the DRG from patients with painful diabetic peripheral neuropathy.^19^ Transcriptional responses vary substantially across different nerve insults,^44^ so it would be of interest to test whether neuronal loss and the subpopulation vulnerability reported in this study are common features across different types of insults.

Using multiple approaches, we assess the naïve mouse L4 DRG to contain approximately 8000 neurons, consistent with a previous estimate,^67^ and observed a frank loss of small-diameter neurons following injury. However, the extent of loss observed using our semiautomated approach was less than that observed using manual techniques.^67^ Two major limitations in this study may explain this discrepancy: First, owing to technical issues, the cleared DRG dataset is unpaired ipsilateral–contralateral which adds larger variability. Second, the analysis method is prone to undercounting deep nuclei. The signal-to-noise is better for superficial nuclei and smaller tissue volumes. Given the reduction in DRG volume after SNI_trans_, nuclei in larger contralateral DRG may be undercounted.

While we made efforts to profile the loss of several molecularly discrete sensory neuron populations, we acknowledge that not all subtypes were profiled. Furthermore, recent single-cell RNA sequencing has given us a more granular appreciation of the heterogeneity of sensory neurons.^42^ Future studies could leverage our experimental approach and new transgenic lines to characterize the loss of neurons in more detail. Such experiments may be pertinent before embarking on molecular or functional profiling of populations post–nerve injury.

### 4.5. Conclusions

In sum, we have provided data from multiple complementary experimental approaches to support the hypothesis that DRG neurons are lost following nerve injury in mice. We describe a substantial loss, which is biased towards specific subpopulations and particularly present in small-diameter nonpeptidergic nociceptive neurons.

## Conflict of interest statement

D.L.B. has acted as a consultant in the last 2 years for AditumBio, Biogen, Biointervene, Combigene, LatigoBio, GSK, Ionis, Lexicon therapeutics, Neuvati, Olipass, Orion, Replay, SC Health Managers, Theranexus, Third Rock Ventures, and Vida Ventures on behalf of Oxford University Innovation. D.L.B. has received research funding from Lilly and Astra Zeneca, and G.A.W. has received research funding from Ono Pharmaceutical. D.L.B. has received an industrial partnership grant from the BBSRC and AstraZeneca. The remaining authors have no conflicts of interest to declare.

Data are available on request to lead contact G.A.W.—gregory.weir@glasgow.ac.uk. Further information and requests for reagents and/or reagents used in this study should also be directed to G.A.W., and we will endeavour to fulfil these.

## Appendix A. Supplemental digital content

Supplemental digital content associated with this article can be found online at http://links.lww.com/PAIN/C84.

## Supplemental video content

Video content associated with this article can be found on the PAIN Web site.

## Supplementary Material
