# Supplementary material for: Peripheral nerve injury results in a biased loss of sensory neuron subpopulations
Source: Pain. 2024 Aug 15;165(12):2863–76. doi: 10.1097/j.pain.0000000000003321 (PMC11562755; doi:10.1097/j.pain.0000000000003321)
Supplement: Supplementary file 2 [file jop-165-2863-s002.pdf]

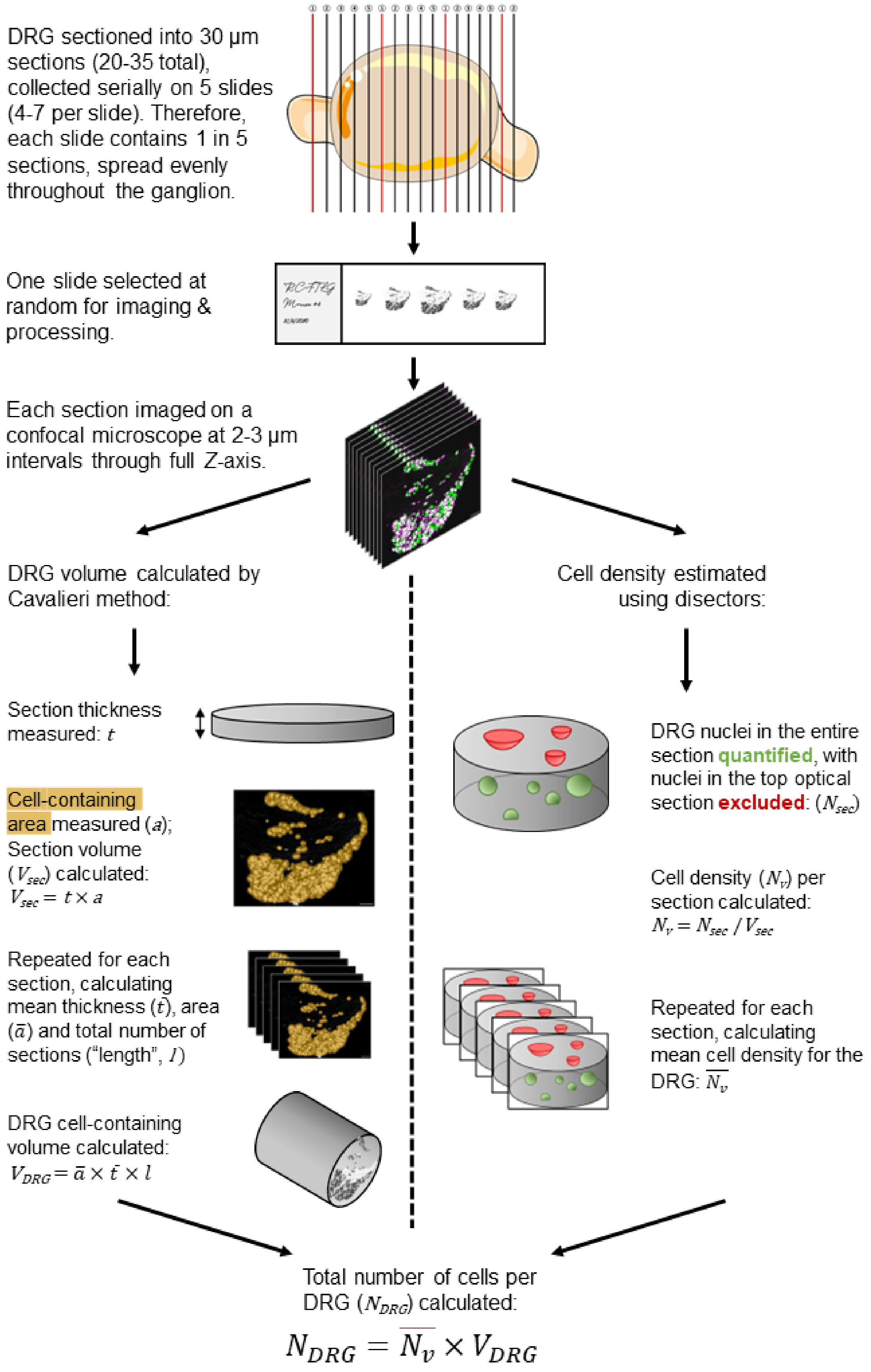

Figure S1 (related to Methods and Figure 1). Schematic of stereological approach to quantify numbers of DRG neurons. Adapted from Coggeshall (1992) [11].

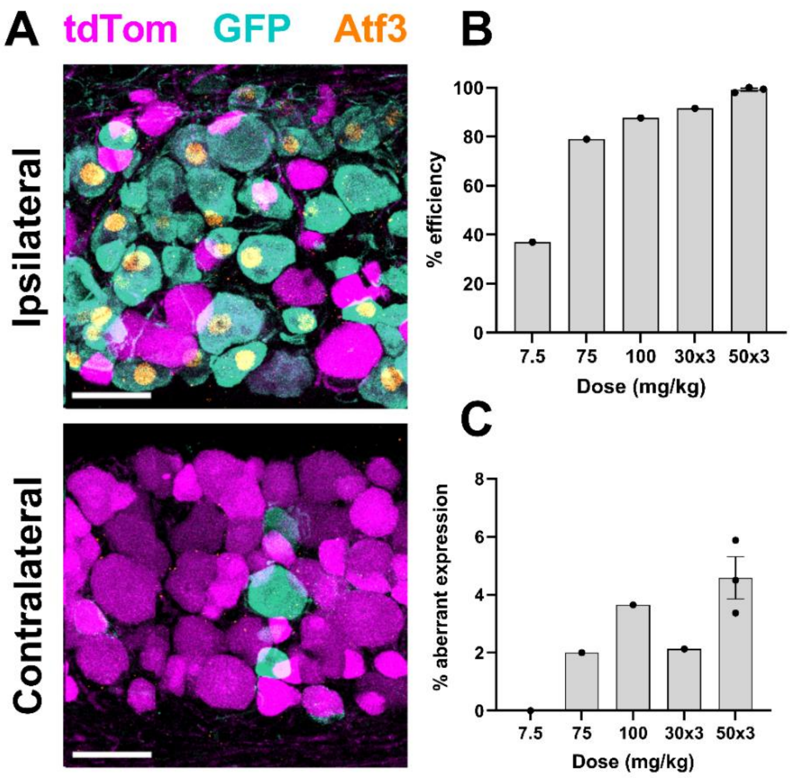

Figure S2 (Data related to Figure 1). Tamoxifen dose-dependently increases recombination efficiency in Avil^{FlpO};Atf3^{CreERT2};RC::FLTG mice. (A) tdTomato (uninjured), GFP (injured) and Atf3 expression 14 days after SNI$_{trans}$ surgery at the ipsilateral and contralateral L4 DRG, following 50 mg/kg tamoxifen at 0, 3 and 7 days post-SNI, the dosing regimen used when collecting data presented in Figure 1. Images are projections of optical sections at 3 µm intervals through the entirety of 30 µm-thick tissue sections. Scale bars = 50 µm. (B) Percentage efficiency of Atf3 recombination at the ipsilateral DRG (i.e., the percentage of Atf3-expressing neurons that co-express GFP) at the initial pilot dosing regimens tested for efficiency of recombination. *n* = 1-3 mice. (C) Quantification of aberrant expression of GFP at the contralateral DRG (i.e., the percentage of neurons expressing GFP).

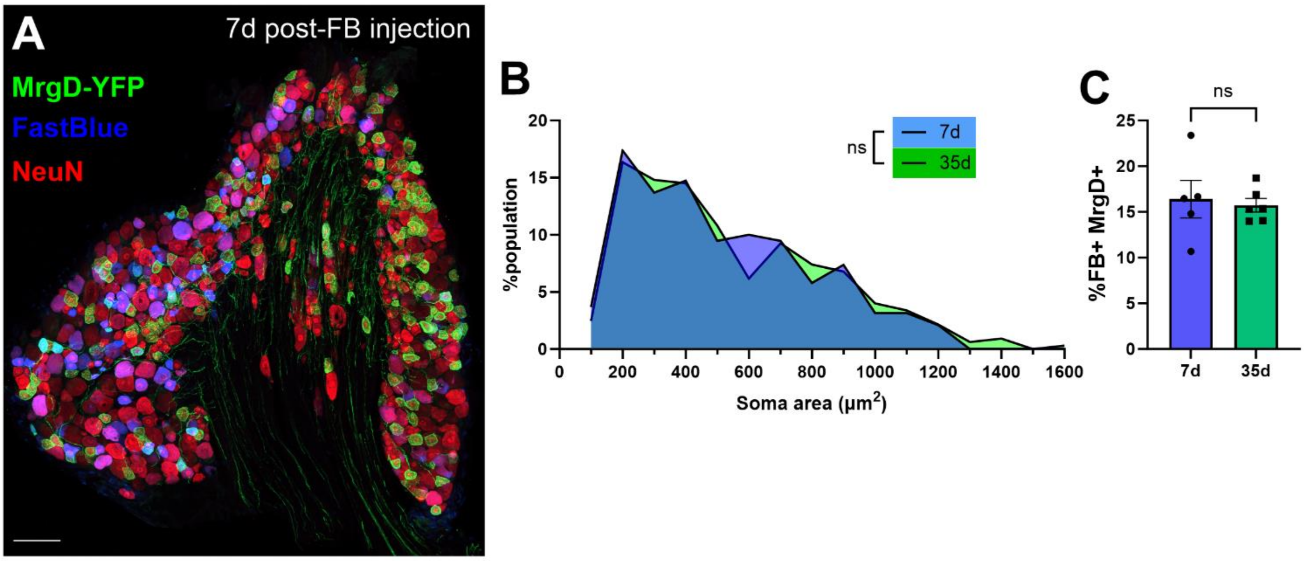

Figure S3 (related to Figures 2 to 4). FastBlue (FB) uptake is equal at 7- and 35-days post-injection, i.e., the potential for reduced FB uptake at 7D vs 35D after injection is not responsible for differing populations of FB-filled cells observed in the ipsilateral versus contralateral DRG following SNI_{trans}. (A) FB labelling and MrgD-YFP and NeuN expression in the L4 DRG of a $MrgD^{CreERT2}$;Ai32 mouse 7 days post-FB injection. Image is a projection of optical sections in the Z axis at 3 µm intervals through the entirety of a 30 µm tissue section. (B) Population distributions of FB-labelled neurons 7- or 35-days after FB injection. Kolmogorov-Smirnov test: D = 0.057, P = 0.83, n = 191-324 neurons from 5-6 mice. (C) Quantification of the percentage of FB-labelled neurons in the L4 DRG that are MrgD-YFP+ 7- or 35-days post-FB injection. 35-day data is pooled from the DRGs contralateral to SNI_{trans} or SNI_{crush} presented in Figure 3G. Unpaired t-test; t_9 = 0.34, P = 0.75, n = 5-6 mice.

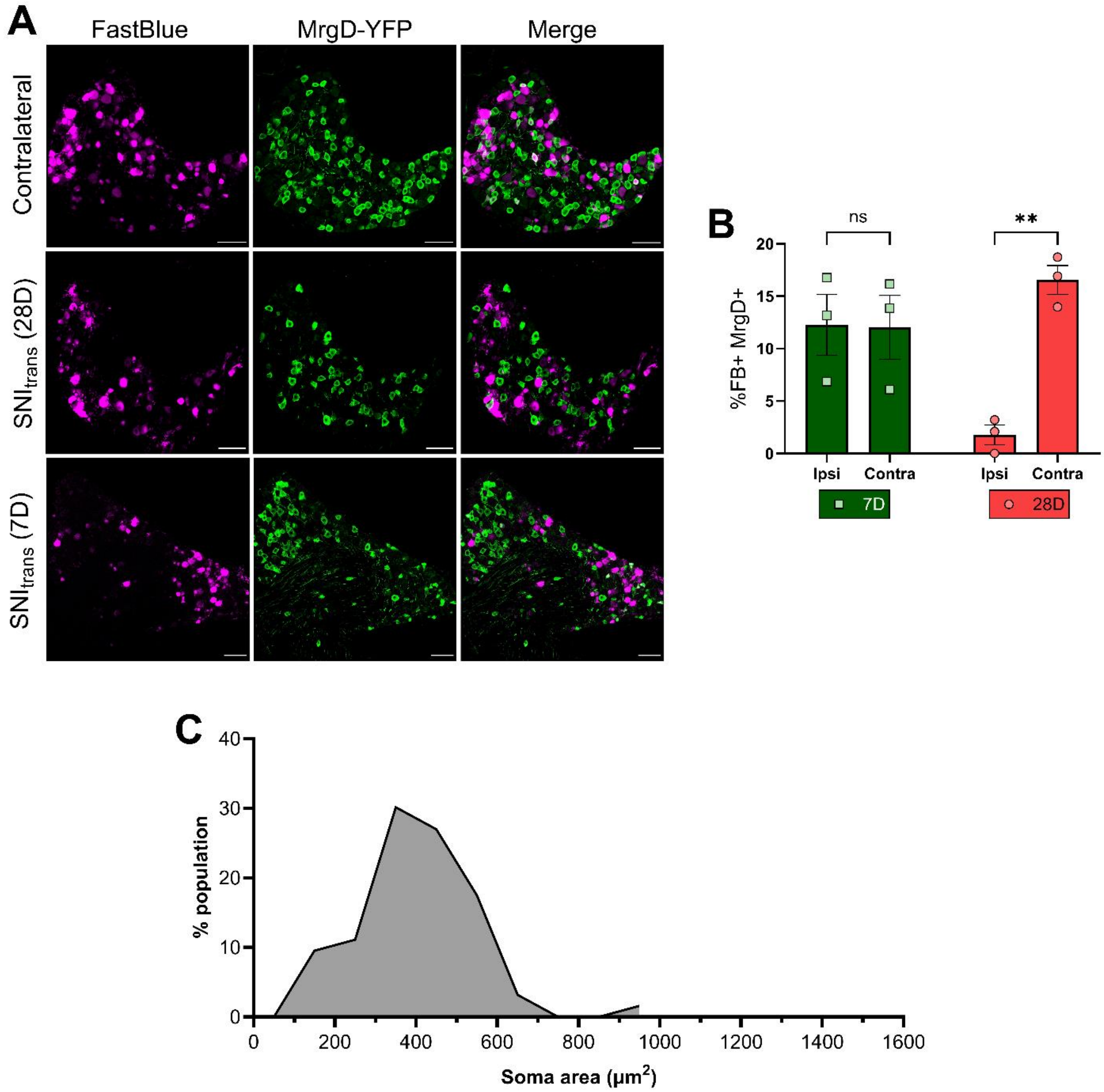

Figure S4 (related to Figure 3). Non-peptidergic neuron death does not occur until after 7d post-nerve injury. (A) FB labelling and MrgD-YFP expression in $MrgD^{CreERT2}$;Ai32 L4 DRGs 7- or 28-days after SNI_{trans} surgery, or contralateral to injury. 28-day post-SNI_{trans} data also shown in **Fig. 3**. Scale bars = 100 µm. (B) Quantification of the percentage of FB-labelled neurons that are MrgD-YFP+. 2-way RM ANOVA; Timepoint x Side interaction: $F_{1,4}$ = 51.3, *P* = 0.002; Šídák's multiple comparisons tests: ** *P* < 0.01; *n* = 3 mice. (C) Quantification of cross-sectional area of FB-labelled, MrgD-YFP^{+} DRG neurons contralateral to SNI_{trans}. *n* = 63 neurons from 3 mice. A rigorous quantification of population distribution at 28d after SNI_{trans} was not possible due to the low number of surviving MrgD-YFP^{+} neurons.

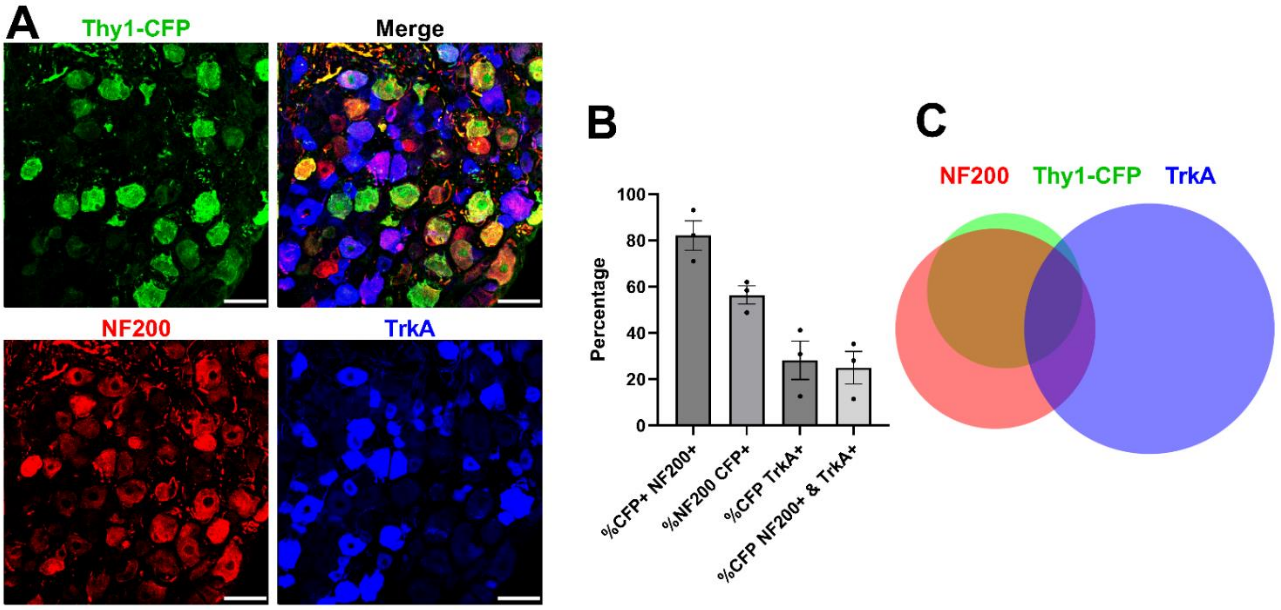

Fig S5 (related to Figure 4). Thy1-CFP is expressed by a heterogenous population of myelinated afferents. (A) expression of Thy1-CFP, NF200 (expressed by myelinated afferents) and TrkA (expressed by a majority of peptidergic afferents) in a lumbar DRG. Image is a projection of optical sections in the Z axis at 3 µm intervals through the entirety of a 30 µm tissue section. (B) Quantification of overlap of expression. *n* = 3 mice. (C) Quantitative Venn diagram illustrating overlap of expression; *n* = 1503 neurons from 3 mice.

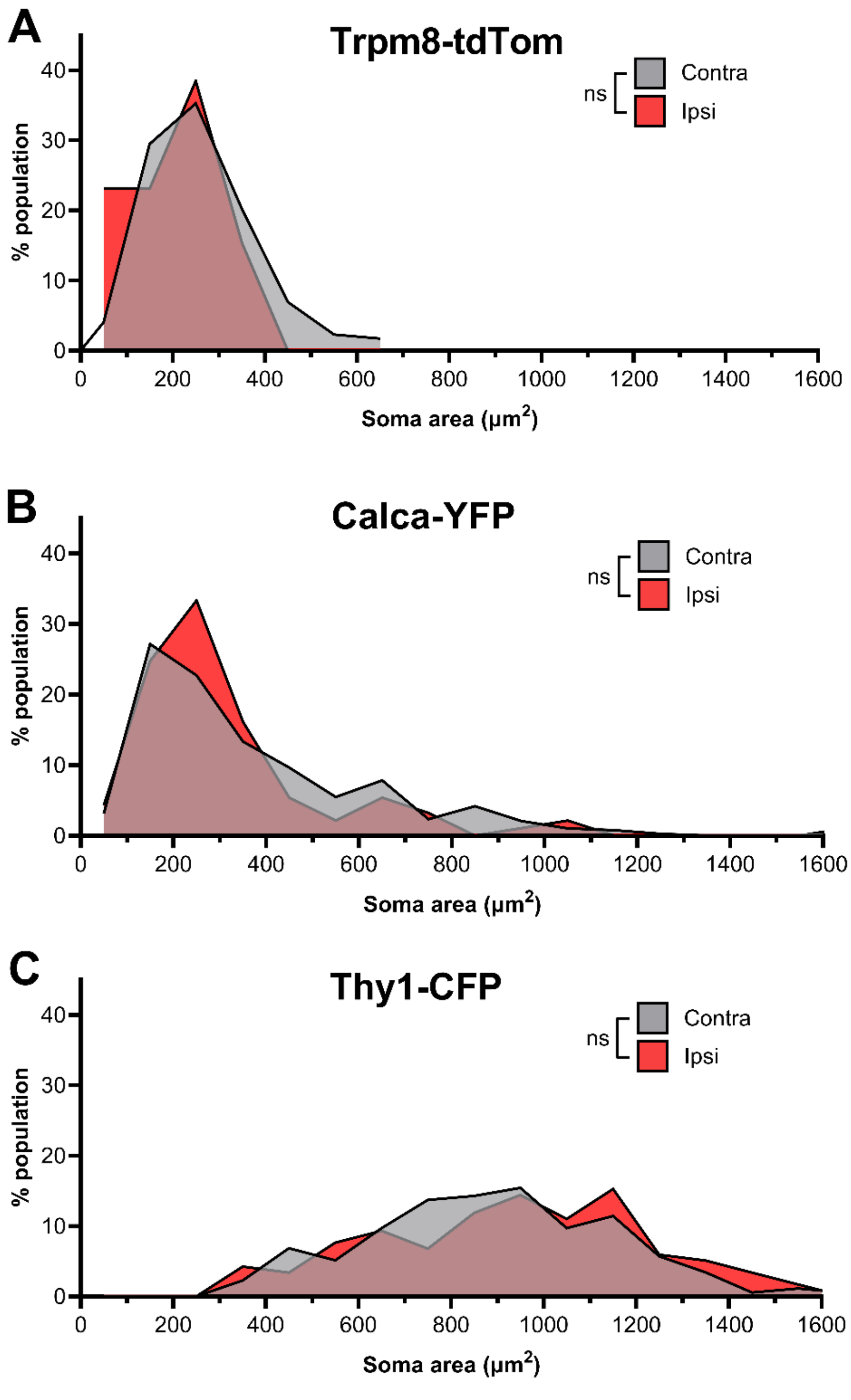

Figure S6 (related to Figure 4). SNI_{trans} does not alter the cell size population distributions of Trpm8-tdTom, Calca-YFP or Thy1-CFP-expressing DRG neurons. Quantification of cross-sectional area of FastBlue labelled neurons in L4 DRG ipsilateral and contralateral to SNI_{trans}, co-labelled with Trpm8-tdTom (A), Calca-YFP (B) or Thy1-CFP (C), 28d after SNI_{trans} surgery. Kolmogorov-Smirnov tests of cumulative distributions; Trpm8-tdTom: D = 0.32, *P* = 0.17, *n* = 13-73 neurons from 3 $Trpm8^{FlpO}$;RC::FLTG mice; Calca-YFP: D = 0.15, *P* = 0.09, *n* = 93-83 neurons from 3 $Calca^{CreERT2}$;Ai32 mice; Thy1: D = 0.13, *P* = 0.17, *n* = 118-175 neurons from 4 Thy1-CFP mice.

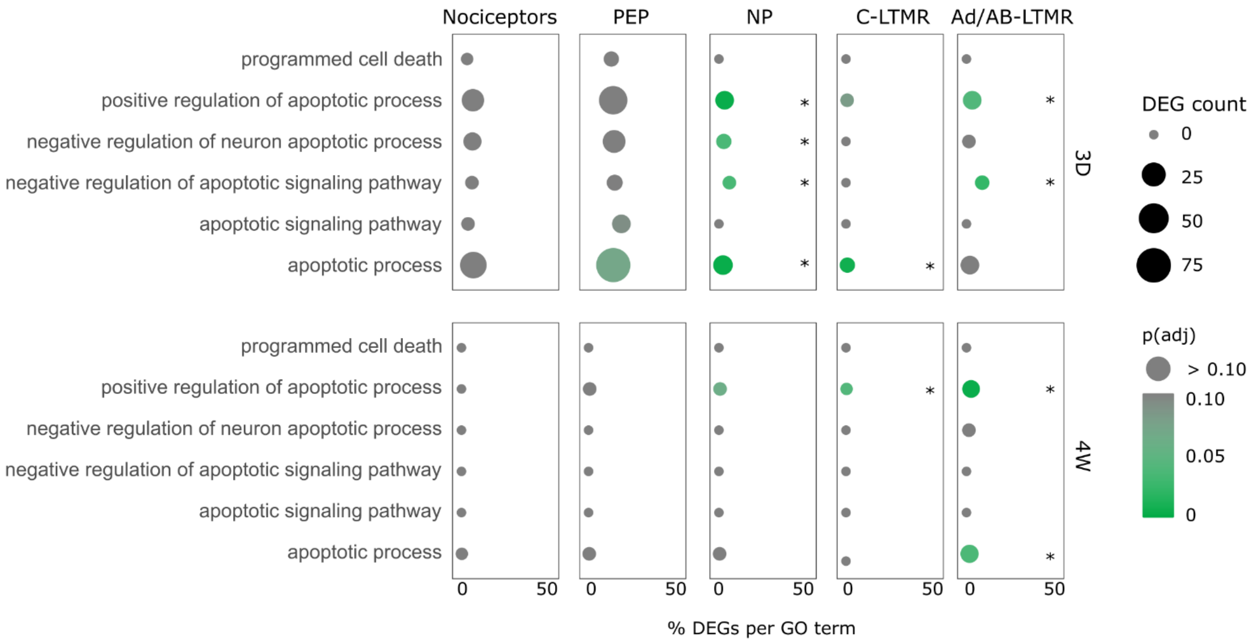

Figure S7 (related to Figure 4). Non-peptidergic neurons show an overrepresentation of cell death pathways 3 days after SNI$_{\text{trans}}$. Gene Ontology (GO) term analysis for cell death pathways across DRG subtypes after SNI (Barry et al., 2023). Five subpopulations were included at 3 days (3D) and 4 weeks (4W) after surgery: general nociceptors (labelled with Scn10a^{CreERT}), peptidergic nociceptors (PEP, Calca$^{\text{CreERT2}}$), non-peptidergic nociceptors (NP, Mrgprd$^{\text{CreERT2}}$), C-LTMRs (Th$^{\text{CreERT2}}$), and Aß-RA (rapidly adapting) + Aδ-LTMRs (Aδ/Aβ-LTMR, Ntrk2$^{\text{CreERT2}}$;Avil^{FlpO}). Significantly over-represented terms ($P < 0.05$) per population and timepoint are represented with an *. DEG, differentially expressed genes.

Supplemental video 1.mp4

Supplemental video 1 (related to Figure 2E-H). Whole DRG TDP-43 expression and nuclear spot profiles. Representative 3D rendering of TDP-43 profiles and corresponding nuclear spot profiles following Imaris-based spot detection feature.
